# Attachment Styles, Perceived Stress and Social Support in a Malaysian Young Adults Sample

**DOI:** 10.5334/pb.320

**Published:** 2016-03-01

**Authors:** Siamak Khodarahimi, Intan H. M. Hashim, Norzarina Mohd-Zaharim

**Affiliations:** 1 Nursing Department, Eghlid Branch, Islamic Azad University, Eghlid, Iran; 2 Universiti Sains Malaysia, MY

**Keywords:** Attachment style, self-perceived stress, social support, gender, ethnicity, religion

## Abstract

The purpose of this research was to examine the validity of an adult attachment style questionnaire, to understand the relationships between the type of attachment style in relation to self-perceived stress and social support, and to investigate the influence of gender, ethnicity and religion on the above constructs. The participants were 308 university students from Malaysia. A demographic questionnaire and three self-report inventories were administrated in this study. The data indicated that the Relationship Scales Questionnaire (RSQ) is a multidimensional construct with nine factors: “dismissing,” “preoccupied with romance,” “preoccupied with close relationships,” “fearful,” “preoccupied with dependency,” “secure emotional,” “comfortable depending,” “preoccupied with mistrust” and “mutual secure.” Different attachment styles were positively or negatively correlated at a significant level with perceived stress and social support. Attachment styles were explained by 20 and 33% of the total variance in self-perceived stress and perceived social support, respectively. There were significant gender, ethnic and religious differences in attachment styles, perceived stress and social support.

## Introduction

Attachment theory focuses on parent-child bonding and postulates that attachment to a caregiver is part of the fundamental survival system. The original theory of Bowlby (1969) defined two types of internal working models, one pertaining to the mental representation of “self,” and one referring to “others.” Bowlby (1969, 1988) proposed that there is an evolutionary basis to attachment, which allows people to perceive and respond to the social environment in an efficient way. He also suggested that attachment styles of adults towards their child included responding with sensitivity and properly to the child’s desires and needs. This may influence interpersonal interactions of the child later on in his or her life. The attachment theory of Bowlby suggests that the child’s relationship with their mother can determine interpersonal performances in terms of their social, emotional and cognitive development in adulthood. Bowlby (1973) stated that individuals create their social environments in ways which confirm their cognitive working models and create the continuity of attachment patterns across their development.

The four-category model of attachment of Bartholomew and Horowitz (1991) is based on both the cognitive working model of the self and the others. These categories include “anxious-ambivalent” and “avoidant” categories of adult attachment. Bartholomew (1990) divided the avoidant category into two distinct categories which reflect differing behavior patterns, thus resulting in a total of four categories labeled: “secure,” “fearful” “preoccupied” and “dismissing” (Bartholomew & Horowitz, 1991). This model suggests that those adults who build up a positive model of other individuals as being potentially accessible and encouraging and themselves as worthy of approval and support can be categorized as securely attached. Individuals with a secure attachment style are supposed to have had early childhood care giving experiences that were reliable, helpful, and approachable, and they are predisposed to be more successful in building supportive and positive relationships. Individuals with a secure attachment style grow a sense of confidence due to caregivers who take action emotionally and thus increase competence to care and demonstrate more positive emotions towards others in social and interpersonal relationships. Individuals with a fearful attachment style have a strong mistrust of others and a view of the self as unlovable and undeserving of care. The preoccupied attachment style can be defined as a mixture of a negative “self-model” and a positive “other” model. Dismissive attachment is a combination of high self-esteem and negative attitude towards others. Bartholomew and Horowitz (1991) showed that these four prototypic attachment patterns are representations of a person’s self-image and image of others in both positive and negative ways in social relations, particularly in young adulthood. The four-category model of attachment predicts that a secure and insecure attachment style has a different relationship with the perception of both stress and social support in interpersonal relationships toward people. Studies showed that healthy attachment working models lead to the formation of healthy relationships during adulthood (Davila, Karney, & Bradbury, 1999; Griffin & Bartholomew, 1994a, 1994b; Mikulincer & Arad, 1999).

However, the current conceptualizations show attachment theory as a culture-sensitive framework influenced by observational instruments and middle-class assumptions in Western cultures. The question remains whether similar results would be obtained in rural eco-social environments in non-Western cultures (Keller, 2013; Mesman & Emmen, 2013). Therefore, it is essential to investigate how a culture can influence the factorial composition of attachment style; and how attachment style will be associated to stress and social support, as well as the role of demographics such as gender, ethnicity and religion and age herein.

### Attachment Style and Culture

Hazan and Shaver (1990) suggested that a person’s approach to his or her family, friendship, work and interpersonal settings would reflect the traits present in the different attachment styles. An attachment figure also provides a *secure base* for exploration in a culture, allowing a person to pursue personal aspirations in a safe and effective way (Bowlby, 1988; Feeney, 2004, 2007). Attachment styles are stable and are the internal working frameworks which guide people’s quality and quantity of relationships with others, and eventually their social functioning (Cassidy & Shaver, 1999; Hazan & Shaver, 1987), social competencies (Mallinckrodt, 2000), psychological adjustment (Cooper, Shaver, & Collins, 1998), and relevant attitudes (Hofstra, Van Oudenhoven, & Buunk, 2005; Van Oudenhoven & Hofstra, 2006). Therefore, it can be speculated that the cognitive models of attachment may have culturally-bound contexts. Research has shown many cross-cultural differences in proportional ratios of different attachment styles among children and adults (Sprecher et al., 1994). These differences could portray the cultural differences in child-rearing practices and its related values (Keller et al., 2004). Furthermore, these internal working models might be influenced by cultural changes in the core aspects of adult interpersonal relationships (Inglehart, 2003). For instance, cross cultural studies have demonstrated that people in collective cultures show a higher tendency to evaluate the self in terms of interconnectedness and the value they will provide to others, than those from individualistic cultures (Markus & Kitayama, 1991; Kitayama, Markus, Matsumoto, & NoRSQakkunkit, 1997). In addition, insecure attachments have been found to be linked to a harsh environment and economic hardships (Schmitt, 2003), whereas preoccupied attachment often co-occurred with high rates of collectivism (Schmitt et al., 2004). Since Malaysia has a general collective culture, this study suggests that attachment styles may have a different multifaceted structure in a Malaysian sample.

### Attachment Style, Perceived-Stress and Social Support

Many studies suggest that there are strong relationships between stress and attachment styles in Western cultures (Craft, Serovich, McKenry, & Lim, 2008; Gormley & Lopez, 2010; Neff, Karney, 2009). According to Collins, Guichard, Ford, and Feeney (2004) care giving is a process which is probably triggered when a person has to cope with a stressor, or when individuals seek help, or clearly could benefit from help. According to Collins and colleagues (2004) support-seeking is well regulated around an empathic stance toward another person. The responsiveness theme incorporates generous intentions and helps the person to feel loved, understood, and cared for (Reis & Shaver, 1988).

Moreover, attachment theory helps scholars understand the development of individual differences in support-seeking and the perception of attainable support in a society. According to Bowlby (1973), these individual differences are the result of a history of interactions with attachment figures, which begin in infancy; the mental representations of these interactions form the *internal working models* of self and others, and relationships. Overall, studies on attachment and perceptions, and expectations of stress and social support incorporate a theory-based prediction that insecure people are more likely to appraise others’ responsiveness negatively (Mikulincer & Shaver, 2009). Indeed, many studies have shown that people with high levels of secure attachment appraise stressful events more optimistically. They attain higher scores on the measures of intimacy, trust, prosocial behavior and relationship satisfaction (Mikulincer & Shaver, 2007). Therefore, we expect that attachments styles as an intrapsychic mechanism influence the perception of stress and social support among young adults in general.

### The Present Study

This study’s predictions are based on attachment theories. Our main aim is to explore the role of culture in attachment styles (Dutton, Starzomski & Ryan, 1996; Collins & Read, 1990; Hazan & Shaver, 1987; Griffin & Bartholomew, 1994a, 1994b; Keller, 2013; Mesman & Emmen, 2013; Simpson, Rholes, & Nelligan, 1992; Waskowic & Chartier, 2003). This study is purposed to evaluate the construct and concurrent validity of the Relationship Scales Questionnaire (Griffin & Bartholomew, 1994a, 1994b; RSQ) in a Malaysian student sample; and to investigate how attachment styles are related to perceived stress and social support, taking into account demographics such as gender, ethnicity and religion and age in a Malaysian student sample. This study is important because there is a lack of evidence about the role of culture on attachment styles in Malaysia. Within a culturally-bound perspective on attachment styles (Keller, 2013; Mesman & Emmen, 2013), this study suggests that Malaysian culture can be categorized as collectivistic more than individualistic. However, within Malaysian culture itself, there are subcultures which have different ethnicities and religions. Each group may represent a certain unique way of life. In line with Griffin and Bartholomew (1994a, 1994b), Keller (2013) and Mesman and Emmen (2013), this study suggests that attachment styles are expected to vary in Malaysian culture. It is also speculated that secure and insecure attachment styles are two basic dimensions, as per Bowlby’s theory, but may take on different forms in this culture. The first hypothesis is that Relationship Scales Questionnaire (RSQ) would have a different multifaceted structure. The second hypothesis is that attachment style would have significant relationships to perceived stress and social support. The third hypothesis is that attachment style would relate to perceived stress and social support. The fourth hypothesis is that gender, ethnicity and religion would have significant influences on attachment style, perceived stress and social support.

## Method

### Participants

The participants were 308 undergraduate students (22% male) of a public university in Malaysia. The means (and standard deviations) of age for males and females were 22.35 (*SD* = 3.36) and 21.85 (*SD* = 1.31), respectively. The sample incorporated various ethnic groups that include Malay (*n* = 152), Chinese (*n* = 91), and others (*n* = 65). Also, the sample incorporated three religions; Islam (*n* = 210), Christian (*n* = 19), and Buddhist (*n* = 79). Participants were selected from those taking a psychology course in the university; they were given extra-credits as an incentive to participate in the study. They were from various departments in the university and from various years of study. After informed consent was acquired, participants completed a questionnaire containing sections on demographic information, attachment style, perceived stress, and social support.

### Instruments

The demographic questionnaire included items on age, gender, religion, ethnicity, major and school of studies, marital status, order of birth, number of siblings, and family size. The three inventories used were: (1) the Relationship Scales Questionnaire (RSQ), (2) the Chinese College Stress Scale (CCSS), and (3) the Multiple Perceived Social Support Scale (MPSSS).

*Relationship Scales Questionnaire* (*RSQ;* Griffin & Bartholomew, 1994a, 1994b).

The RSQ is a 30-item scale that provides a continuous measure of one’s typical subjective style in close relationships and assesses four dimensions: secure, fearful, dismissing and preoccupied. The “Secure” style indicates that the individual has positive views of self and others. The “Fearful relationship” style indicates negative perceptions of self and others. The “Preoccupied” style is defined by a negative view of self and a positive view of others. The “Dismissing” style relates to a high level of self-confidence and negative views of others. This measure has been reported to show strong convergent and divergent validity (Griffin & Bartholomew, 1994a, 1994b). The RSQ’s internal consistency using Cronbach’s alpha in the present study was = .73.

*Chinese College Stress Scale (CCSS, Li & Lin, 2005)*. The CCSS has 34 items and includes three subscales: personal hassles (daily stressors, 19 items), academic hassles (learning and examination stressors, 11 items), and negative life events (personal and academic events, 4 items). Items are completed on a Likert scale ranging from 1 (*strongly low*) to 4 (*strongly high*). Using Cronbach’s alpha, the internal consistency estimates were .84 for the academic hassles subscale, .88 for personal hassles, and .83 for negative life events. The entire 30-item CCSS had an internal-consistency estimate of .92.

*Multiple Perceived Social Support Scale* (MPSSS; Zimet et al., 1988). The MSPSS is a 12-item scale which was developed to assess perceived social support from family, friends, and significant others. The MSPSS uses a 7-point Likert scale (1 = *strongly disagree* to 7 = *strongly agree*). The MSPSS produces three scores. The MSPSS’s reliability and validity were confirmed in several studies (Canty-Mitchell, & Zimet, 2000; Zimet et al., 1988, Zimet, Powell, Farley, Werkman, & Berkoff, 1990). The MSPSS’s internal consistency using Cronbach’s alpha in the present study was .91.

## Results

To examine the first hypothesis, a factor analysis was conducted to evaluate the multidimensional nature of the RSQ and its construct validity in a non-clinical sample. Principal factor analysis, with oblimin rotation was used to determine the construct validity, considering all factors that had an Eigenvalue higher than 1. Factor analysis specification was satisfactory; KMO = .75, Bartlett’s Test of Sphericity = 2.55, df = 436, *p* = .0001, Rotation Sums of Squared Loadings = 61.25. Table 1 shows the loadings of the items of the rotated solution, with loadings higher than .30 for 27 items. Ten iterations were run and 4 items were rejected in this process (i.e., 4, 6, 9, and 28 in the original version). Eigenvalues for the nine factors ranged from 3.38 to 17.25. These factors explained 61.25% of the total variance. These factors were interpreted as “dismissing,” “preoccupied with romance,” “preoccupied with close relationships,” “fearful,” “preoccupied with dependency,” “secure emotional,” “comfortable depending,” “preoccupied with mistrust,” and “mutual secure” (Table 2). The RSQ’s internal reliabilities by Cronbach’s alpha were greater than .83 for all factors and .86 for the total scale.

**Table 1:** Rotated Component Matrix of Relationship Scales Questionnaire (RSQ).

Items	1	2	3	4	5	6	7	8	9

1	.087	–.395	–.313	–.203	**.628**	–.290	.156	–.366	–.320
2	–.130	–.223	–.570	–.332	**.700**	–.208	–.038	–.096	.020
3	–.200	–.458	–.362	.197	.031	**.739**	–.302	.127	–.069
5	–.304	–.404	–.354	**.649**	.107	–.278	–.294	–.087	–.286
7	–.310	–.269	–.086	**.510**	.099	–.288	.083	–.073	.170
8	–.473	–.411	**.535**	–.041	.159	.109	.017	–.064	.119
10	–.513	–.324	–.224	–.338	–.143	–.240	**.340**	.202	–.034
11	–.210	**.811**	–.336	–.289	–.191	.173	.247	–.248	.126
12	–.166	–.414	.210	.076	–.136	.145	–.461	**.422**	–.336
13	.110	.079	–.132	**.601**	–.059	.026	.133	–.205	–.330
14	–.078	–.058	**.689**	–.367	.218	–.117	–.317	–.541	–.208
15	–.127	.149	**.733**	–.367	.119	–.226	–.212	–.230	–.219
16	–.309	–.124	**.488**	–.162	–.437	–.263	–.355	.236	–.227
17	–.235	–.341	.146	–.409	–.198	–.243	–.235	**.653**	–.318
18	**.520**	.154	.122	–.386	.131	–.083	–.180	–.268	–.212
19	**.606**	–.347	–.314	–.189	–.207	.124	–.154	–.354	.118
20	**.661**	–.429	.093	–.336	.015	–.125	–.237	.060	–.178
21	–.138	**.832**	–.252	.218	–.282	–.221	–.068	–.327	.120
22	**.654**	–.322	.158	–.185	–.458	–.074	–.035	–.209	–.230
23	–.197	**.558**	–.306	–.262	–.309	.046	.059	–.344	.110
24	**.613**	–.206	.049	–.209	–.178	–.156	–.052	–.059	–.060
25	.177	.012	–.366	.163	.144	–.375	–.086	**.631**	–.278
26	.136	–.257	.240	–.028	**.620**	–.029	–.049	–.266	.215
27	.162	–.348	**.377**	–.058	–.256	–.364	.099	–.276	.715
29	–.295	**.422**	.026	.056	–.178	.120	–.234	.087	–.026
30	–.496	.025	.138	–.156	.144	.126	.129	–.483	**.617**

**Table 2:** Factors and Items.

Factors	Items	Cumulative %

1. Dismissing	18, 19, 20, 22, 24	17.25
2. Preoccupied with Romance	11, 21, 23, 29	28.71
3. Preoccupied with Close relationships	8, 14, 15, 16	34.94
4. Fearful	5, 7, 13	40.77
5. Preoccupied with Dependency	1, 2, 26	45.67
6. Secure Emotional	3	50.34
7. Preoccupied with Mistrust	10	54.21
8. Preoccupied with Mistrust	12, 17, 25	57.86
9. Mutual Secure	27, 30	61.25

To test the second hypothesis, a correlational analysis was computed to evaluate the relationships between attachment style, perceived stress and social support. Analysis indicated that perceived stress was positively correlated at a significant level with “dismissing,” “preoccupied with romance,” “preoccupied with close relationships” and “fearful” attachment styles. In contrast, perceived social support and its subscales were negatively associated with” dismissing,” “fearful,” “preoccupied with dependency” and “preoccupied with mistrust” attachment styles. In addition, there was a significant negative relationship between perceived social support from friends and “preoccupied with romance” attachment style. The MPSSS was positively correlated with the “secure emotional” and “mutual secure” attachment styles. The present findings indicated significant positive correlation coefficients between “dismissing,” “preoccupied with romance,” “preoccupied with close relationships,” “fearful,” “preoccupied with dependency,” “comfortable depending” and “preoccupied with mistrust” attachment styles; all of them yielded one cluster in the attachment style called “insecure.” “Mutual secure” attachment had a significant positive correlation with “secure emotional” attachments, and had significant negative relationships with “preoccupied with romance,” “preoccupied with close relationships” and the “fearful” styles (Table 3).

**Table 3:** Attachment Styles, Perceived Stress and Social Support Correlations.

Variables	2	3	4	5	6	7	8	9	10	11	12	13	PSST

1. Dismissing	.261^**^	.203^**^	.405^**^	.273^**^	–.035	.182^**^	.295^**^	–.012	.196^*^	–.231^**^	–.194^**^	–.168^**^	–.250^**^
2. Preoccupied with romance		.414^**^	.207^**^	–.051	–.137^*^	.256^**^	.257^**^	–.134^*^	.227^**^	.095	–.139^*^	.024	–.016
3. Preoccupied with close relationships			.106	–.020	–.198^**^	.182^**^	.304^**^	–.251^**^	.236^**^	.106	–.039	.055	.048
4. Fearful				.190^**^	–.051	.016	.323^**^	–.168^**^	.261^**^	–.235^**^	–.180^**^	–.233^**^	–.263^**^
5. Preoccupied with dependency					–.091	–.153^**^	.138^*^	.016	.092	–.149^**^	.003	–.170^**^	–.123^*^
6. Secure emotional						.072	.013	.328^**^	.135	.311^**^	.254^**^	.268^**^	.354^**^
7. Comfortable depending							.067	.069	.081	–.064	–.099	.017	–.065
8. Preoccupied with mistrust								–.072	.257^**^	–.198^**^	–.115^*^	–.204^**^	–.219^**^
9. Mutual secure									–.147	.341^**^	.187^**^	.406^**^	.381^**^
10. Stress										–.230^**^	–.172^*^	–.176^*^	–.242^**^
11. Society support											.495^**^	.609^**^	.874^**^
12. Friends support												.403^**^	.763^**^
15. Family support													.819^**^

*Note:* *p ≤ .05, **p ≤ .01. PPST = Perceived Social Support Total.

To test the third hypothesis, multiple regression analyses were conducted to evaluate the possible roles of attachment styles (9 factors) in predicting perceived stress and social support in this sample. Findings revealed that attachment styles accounted for 20% of the variance in perceived stress, *R* = *.450, R^2^* = .202, *F* (9,160) = 4.252, *p* < .0001. “Preoccupied with mistrust” had significant positive relationships with perceived stress, but there was a significant negative relationship between “mutual secure” style and perceived stress. Findings showed that attachment styles explained 20% of the variance in social support, *R* = .578, *R^2^* = .335, F (9,286) = 15.234, *p* < .0001. “Secure emotional” and “mutual” secure styles had significant positive relationships with social support, but there was also a significant negative relationship between “preoccupied with mistrust” style and social support (Table 4).

**Table 4:** Predictors for Perceived Stress and Social Support in the Total Sample and by Attachment Styles.

Dependents	Predictors	*R*	*R^2^*	*β*	*t*	*p*

Perceived-stress	Dismissing	.450	.202	–.017	–.182	.856
	Preoccupied with romance			.103	1.242	.216
	Preoccupied with close relationships			.125	1.463	.145
	Fearful			.113	1.271	.206
	Preoccupied with dependency			.033	.421	.674
	Secure emotional			.196	2.402	.018
	Comfortable depending			.022	.285	.776
	Preoccupied with mistrust			.192	2.052	.042
	Mutual secure			–.231	–2.780	.006
Perceived-social support	Dismissing	.578	.332	–.095	–1.593	.112
	Preoccupied with romance			.030	.526	.599
	Preoccupied with close relationships			.074	1.275	.203
	Fearful			–.062	–1.057	.291
	Preoccupied with dependency			–.011	–.199	.843
	Secure emotional			.274	5.159	.000
	Comfortable depending			–.091	–1.742	.083
	Preoccupied with mistrust			–.275	–4.479	.000
	Mutual secure			.216	3.852	.000

The fourth hypothesis of this study is that gender, ethnicity and religion are significantly related to attachment style, perceived stress and social support. Initially, the multiple regression models were computed to evaluate the possible roles of gender, religion and ethnicity as independent variables in predicting these constructs. Findings proved that gender, religion and ethnicity explained 6% of the variance for attachment style, *R* = .259, *R^2^* = .067, *F* (3, 238) = 5.616, *p* < .001. Results affirmed that gender, religion and ethnicity could also explain 5% of the variance for perceived stress, *R* = .234, *R^2^* = .055, *F* (3,142) = 2.672, *p* < .05. Results indicated that gender, religion and ethnicity explained 4% of the variance for perceived social support; *R* = .220, *R^2^* = .048, *F* (3,243) = 4.050, *p* = .008.

Additionally, a t-test for independent groups was conducted to evaluate the effect of gender, and two ANOVAs were calculated for religion and ethnicity differences in the aforementioned independent variables (Table 5). Findings indicated that males scored higher in “dismissing,” *t* (302) = 4.32, *p* < .0001, and “preoccupied with dependency,” *t* (303) = 3.06, *p* < .002, than females; and females had a significantly higher “secure emotional” style, *t* (303) = –2.07, *p* < .039, than males. There were significant ethnic differences in “dismissing,” *F* (2, 247) = 7.84, *p* < .001; “preoccupied with close relationships,” *F* (2, 248) = 3.08, *p* < .04; “secure emotional,” *F* (2, 250) = 4.24, *p* < .015; and “comfortable depending,” *F* (2, 250) = 6.09, *p* < .003, attachment styles. Moreover, there were significant ethnic differences in perceived stress, *F* (2, 142) = 4.06, *p* < .019, family support, *F* (2, 244) = 6.29, *p* = .002; and the total perceived social support, *F* (2, 243) = 4.24, *p* < .015. Findings indicated significant religious differences in “dismissing,” *F* (2, 304) = 8.72, *p* < .0001; and “comfortable depending” styles, *F* (2, 307) = 7.01, *p* < .001; family support, *F* (2, 301) = 5.48, *p* < .005, and total perceived support, *F* (2, 300) = 3.63, *p* < .02.

**Table 5:** Attachment Style, Perceived Stress and Perceived Social Support Means and Standard Deviations by Gender, Ethnicity and Religion.

Variables	Gender				Ethnicity						Religion					
	Male		Female		Malay		Chinese		Others		Islam		Buddhist		Christian	
	M	SD	M	SD	M	SD	M	SD	M	SD	M	SD	M	SD	M	SD

Dismissing	13.68	4.04	11.57	3.37	11.21	3.21	13.08	3.61	13	5.15	11.46	3.44	13.33	3.55	13.73	4.39
Preoccupied with romance	11.71	3.89	11.93	3.66	11.35	4.10	12.22	3.25	13.62	3.06	11.46	14.14	12.01	4.46	12.42	2.81
Preoccupied with close relationships	13.29	3.22	12.40	3.01	12.17	3.21	13.20	2.96	12.87	3.27	12.31	3.15	13.14	3.00	13.22	2.71
Fearful	9.18	2.62	9.03	2.33	9.15	2.43	8.91	2.38	9.50	3.16	9.11	2.40	8.88	2.37	9.31	2.68
Preoccupied with dependency	10.91	2.28	9.92	2.26	10.20	2.24	10.24	2.07	10.25	2.96	10.07	2.33	10.27	2.03	10.63	2.43
Secure emotional	3.10	1.04	3.41	1.10	3.39	1.12	3.2.	1.11	4.37	.74	3.40	1.09	3.12	1.12	3.52	.96
Comfortable depending	2.46	1.06	2.52	2.24	2.17	1.00	3.16	3.32	2.25	1.28	2.23	1.04	3.22	3.54	2.63	1.06
Preoccupied with mistrust	8.91	2.24	8.82	3.57	8.69	4.08	9.00	2.41	7.75	1.83	8.75	3.68	8.89	2.35	9.52	2.48
Mutual secure	6.42	1.67	6.43	1.57	6.55	1.63	6.25	1.50	6.75	2.12	6.52	1.16	6.12	1.52	6.52	1.61
Perceived stress	91.17	18.84	89.25	18.29	93.23	18.91	86.42	17.22	66.50	26.74	91.45	18.91	86.85	16.88	85.61	18.64
Society support	19.07	5.66	22.61	5.04	22.38	5.15	21.08	5.73	24.00	3.89	22.19	5.20	20.62	5.82	22.63	4.77
Friends support	18.48	4.93	20.43	4.50	20.53	4.58	19.69	4.88	21.37	3.33	20.21	4.52	19.48	4.62	19.94	6.09
Family support	21.86	4.26	23.14	4.63	23.45	4.37	21.48	5.14	25.37	1.92	23.40	4.21	21.40	4.96	22.63	5.61
Perceived social support	59.67	12.50	66.22	11.37	66.54	11.61	62.40	12.71	70.75	4.86	65.92	11.48	61.59	12.69	65.21	11.59

*Note*: Male = 67, Female = 241, Malay = 152, Chinese = 91, Others = 8, Muslim = 210, Buddhist = 79, Christian = 19.

Post-hoc LSD tests indicated that Chinese and Other participants had a significantly higher “dismissing” style than Malays. Chinese participants had significantly higher “preoccupied with close relationships” style than Malay and Others. Other participants had significantly higher “secure emotional” attachment than the Malays and Chinese, and Malays and Others had significantly higher “comfortable depending” style than Others. Malay and Chinese participants had significantly higher perceived stress than Others. Other participants had significantly higher family support and total perceived support than Malay and Chinese participants. Muslim participants had significantly lower “dismissing” attachment than Buddhist and Christian participants. Muslim and Christian participants had significantly lower “comfortable depending” style than Buddhist participants. But Buddhist participants had significantly lower family support and total perceived support than Muslims and Christians.

Additionally, to examine possible gender differences and age groups’ interaction, a multivariate analysis of variance (MANOVA) was run with gender, ethnicity and religion and their interactions as independent variables and the attachment style, perceived stress and social support as dependent variables. There were no significant interactions effects between gender, ethnicity and religion for attachment style, perceived stress and social support.

## Discussion

With respect to our first hypothesis, the results from this study collected in a Malaysian sample confirmed that attachment style is a multidimensional construct. We obtained nine factors in which were labeled “dismissing,” “preoccupied with romance,” “preoccupied with close relationships,” “fearful,” “preoccupied with dependency;” “secure emotional,” “comfortable depending,” “preoccupied with mistrust” and “mutual secure.” This finding is in line with many cross-cultural differences in adult attachment style which highlights the possible roles of cultural factors such as child rearing, values, psychological well- being, social competence and social functions of relationships in the emergence of attachment style, all of which manifest themselves in different forms across different cultures (Cassidy & Shaver, 1999; Cooper, Shaver, & Collins, 1998; Hazan & Shaver, 1987; Hofstra, Van Oudenhoven, & Buunk, 2005; Inglehart, 2003; Kitayama, Markus, Matsumoto, & NoRSQakkunkit, 1997; Markus & Kitayama, 1991; Keller, 2013; Keller et al, 2004; Mallinckrodt, 2000; Mesman & Emmen, 2013; Schmitt et al., 2004; Sprecher et al., 1994; Van Oudenhoven & Hofstra, 2006). As Malaysian culture is part of Asian traditions, which in turn encompasses multiple subcultures, it is expected to demonstrate a few different and unique attachment styles due to the differences in child rearing, education, socialization and aspirations towards ideal models of social relationships. In agreement with Bowlby’s theory and Keller’s conceptualization, it seems there are two basic types of attachment: secure and insecure. They, however, have different forms across cultures. In this study, “secure,” “emotional” and “mutual” secure styles are varieties of the secure attachment. “Dismissing,” “preoccupied with romance,” “preoccupied with close relationships,” “fearful,” “preoccupied with dependency,” “comfortable depending” and “preoccupied with mistrust” styles are examples of the insecure attachment.

However, friendliness may be an important cultural construct that exists across subcultures within Malaysia and this is indicated by their emotional orientations toward social and interpersonal relationships. This tendency is reflected as “preoccupied with romance,” “preoccupied with close relationships,” “preoccupied with dependency,” “secure emotional,” “comfortable depending” and “preoccupied with mistrust” in the present study. The above attachment styles could express the importance of emotions for Malaysian young adults in different social settings and various types of relationships. On the other hand, this capability for emotionality in the Malaysian people is often defined by subcultural and traditional forces across their experiences within their families, friendships, work settings and social networks. Consequently, there are a few distinguished schemas and cognitive working models for these attachment styles, and they might transmit from childhood to adulthood by individual socialization within families and social institutions. However, this conceptualization needs further exploration in both clinical and non-clinical samples by techniques such as schema analysis and qualitative research.

The present results with respect to the second hypothesis affirmed that perceived stress was significantly and positively correlated with “dismissing,” “preoccupied with romance,” “preoccupied with close relationships” and “fearful” attachment styles. In contrast, social support and its subscales were negatively related with “dismissing,” “fearful,” “preoccupied with dependency” and “preoccupied with mistrust” styles. Moreover, there was a significant negative relationship between social support from friends and “preoccupied with romance” style. In addition, social support was positively associated with “secure emotional” and “mutual secure” styles. These findings are consistent with previous speculations and attachment theories’ predictions, and they are in line with previous studies that related attachment relationships to perceived stress and supportive seeking behaviors (Bowlby, 1969/1982, 1988; Feeney, 2004, 2007; Gillath, Shaver, & Mikulincer, 2007; Craft, Serovich, McKenry, & Lim, 2008; Gormley & Lopez, 2010; Keller, 2013; Neff, Karney, 2009; Reis & Shaver, 1988; Mikulincer & Shaver, 2009; Griffin & Bartholomew, 1994a, 1994b).

Findings with respect to the third hypothesis supported the role of attachment style in relationship to perceived stress and social support. Results revealed that “secure emotional,” “preoccupied with mistrust” and “mutual secure” styles explained 20% of the variance in perceived stress. The “secure emotional,” “preoccupied with mistrust,” and “mutual secure” styles summation explained 33% of the variance in perceived social support. These findings help to understand the role of attachment styles on perception of social stressors and negative emotions (Keller, 2013; Mesman & Emmen, 2013). Therefore, people with insecure attachment styles demonstrate negative mental processing and vulnerability to stressful events, and they are more reactive towards such situations. Individuals with a secure attachment style have more positive inner faith toward social support in distress settings. Thus, attachments styles could operate as automatic and spontaneous schemas when the individual encounters stressful events and seeks support against threats. In sum, attachment styles may have an influential role in human stress diathesis and resilience, or personal hardiness and perceived support against stress.

With respect to the fourth hypothesis, our findings showed that gender, religion and ethnicity are related to attachment style, perceived stress and social support. Males had significantly higher “dismissing” and “preoccupied with dependency” styles than females, and females had significantly higher “secure emotional” style than males. Findings showed significant ethnic differences in “dismissing,” “preoccupied with close relationships,” “secure emotional” and “comfortable depending” attachment styles, perceived stress, family support, and total social support. Findings indicated significant religious differences in “dismissing” and “comfortable depending” styles, family support and total support. In agreement with the present findings, previous studies showed similar gender differences in attachment style during childhood and similar relationships with family environments (Mikulincer & Florian, 1999; Searle & Meara, 1999). However, the relationship between religiosity and attachment insecurity is not straightforward and well established. For example, Granqvist and Hagekull (2000) pointed out that for some people, religion appears to act as a compensatory factor in the case of attachment. Similarly, Kirkpatrick and Shaver (1992) found that secure attachment to God was related to adult attachment for individuals who rated their childhood maternal attachments as insecure. Therefore, human attachment styles may have a developmental and an evolutional basis, and their manifestations may vary across societal determinants like gender, ethnicity and religion. These three socio-cultural constructs indicate that people develop attachments through their experiences with significant others within social arrangements and institutions. Alternatively, culturally-bound roles and a personal sense of inferiority and insecurity in these societal contexts could create compensatory mechanisms in individual attachment styles during later stages.

## Conclusion

The current research adds to the body of psychological literature by contributing to knowledge with regard to the effects of culture, gender, ethnicity and religion on attachment styles. We showed relationships between attachment style, perceived stress and social support in a youth sample. Nevertheless, this study is somewhat limited because it relies mainly on survey data, application of exploratory factor analysis, and the recruitment of a specific sample of undergraduate students. Future studies might use confirmatory factor analysis (CFA) and may determine whether a 9-factor structure is the best conceptualization in Malaysian culture. Future research might also apply experimental and longitudinal designs with observational instruments to examine these constructs across different cultural samples, both in clinical and non-clinical populations.

## Competing Interests

Authors wish to confirm that there are no known conflicts of interest associated with this publication and there has been no significant financial support for this work that could have influenced its outcome.
